# Employing conservation of co-expression to improve functional inference

**DOI:** 10.1186/1752-0509-2-81

**Published:** 2008-09-22

**Authors:** Carsten O Daub, Erik LL Sonnhammer

**Affiliations:** 1 Department of Cell and Molecular Biology, Karolinska Institutet, 171 77 Stockholm, Sweden; 2 Stockholm Bioinformatics Center, Albanova, Stockholm University, 10691 Stockholm, Sweden; 3 Omics Science Center, RIKEN Yokohama Institute, 1-7-22 Suehiro-cho, Yokohama, Kanagawa 230-0045, Japan

## Abstract

**Background:**

Observing co-expression between genes suggests that they are functionally coupled. Co-expression of orthologous gene pairs across species may improve function prediction beyond the level achieved in a single species.

**Results:**

We used orthology between genes of the three different species *S. cerevisiae*, *D. melanogaster*, and *C. elegans *to combine co-expression across two species at a time. This led to increased function prediction accuracy when we incorporated expression data from either of the other two species and even further increased when conservation across both of the two other species was considered at the same time. Employing the conservation across species to incorporate abundant model organism data for the prediction of protein interactions in poorly characterized species constitutes a very powerful annotation method.

**Conclusion:**

To be able to employ the most suitable co-expression distance measure for our analysis, we evaluated the ability of four popular gene co-expression distance measures to detect biologically relevant interactions between pairs of genes. For the expression datasets employed in our co-expression conservation analysis above, we used the GO and the KEGG PATHWAY databases as gold standards. While the differences between distance measures were small, Spearman correlation showed to give most robust results.

## Background

Elucidating the function of genes and proteins constitutes one of the main challenges in the post-genomic era. Large-scale gene expression measured by microarrays is a valuable and under-exploited data resource to discover functionally coupled genes. Genes with similar expression profiles, for example, tend to code for interacting proteins and by this enabled further hypotheses about the genes and their corresponding proteins functions [1,2].

Various methods are available for the identification of genes that share patterns in their expressional behavior under different experimental conditions. The measure of similarity between the expression profiles of two genes, where similar genes are said to have a smaller distance to each other than less similar genes, constitutes an important parameter toward the recognition of functionally coupled genes. This distance, however, is often chosen on an ad-hoc basis without systematic analysis of its direct impact on the relevance of the detected interactions.

In previous work by others in *S. cerevisiae*, a distance measure evaluation was performed on binarized gene expression profiles followed by clustering [3]. The statistical evaluation of the resulting gene clusters favored Jaccard's similarity, which is only applicable to binary data. In a comparison of different clustering methods, also in *S. cerevisiae*, the Euclidean and Pearson distance measures were found to produce an optimal number of clusters according to Saccharomyces Genome Database annotations found in the GO database [4]. A recent study, also exclusively in *S. cerevisiae*, proposed a set of novel distance measures for expression pattern detection and compared them to the most frequently applied ones [5]. In contrast to previous work that focused on the results of the subsequent clustering, they directly evaluated the detected candidate interactions in terms of their confirmation with experimental interaction data like protein-protein interactions, KEGG pathway membership, promoter co-regulation, and sequence homology. From the joint information content of all these criteria they infer experimentally verified gene associations to which they compared their candidate interactions.

**Figure 1:**
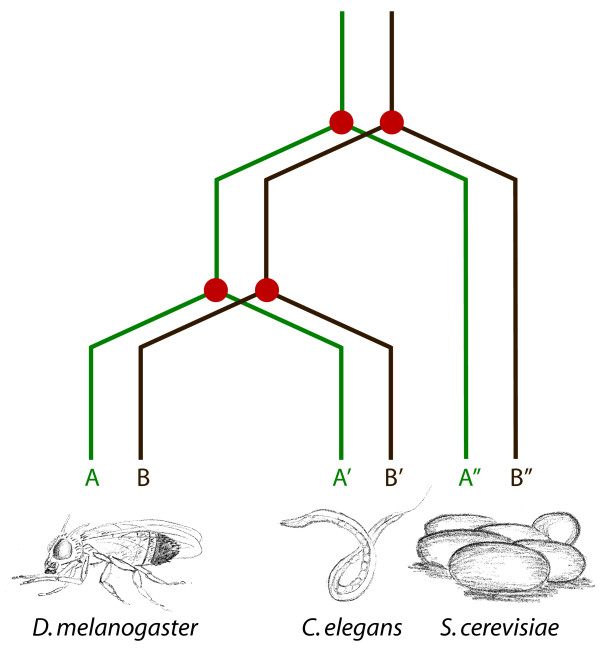
**Schematic representation of orthologous co-regulation**. For two genes A and B in one species, co-expressed genes are more likely to functionally interact than is expected by chance. If the co-expression is conserved, i.e. also found in an orthologous gene pair, A' and B' in another species, where A is orthologous to A' (A and A' arouse from a speciation event, red circle in the figure) and B is orthologous to B', the probability of a functional interaction is increased. This likelihood might be even further increased if the conservation of co-expression occurs across three species at the same time. One might speculate that such conserved co-regulations have an ancient origin and constitute basic functionalities.

The integration of functional genomics data of various types has proven a valuable approach. Gene function prediction was improved by considering co-expression not only in one species, but by taking interactions into account that were found for orthologous gene pairs in another species. One speaks about orthologous co-expression between two genes A and B in one species if the co-expression is also observed between the two genes A' and B' in another species, where A and A' are orthologs to each other and B and B' are also orthologs to each other (Figure 1). This conservation adds reliability to the observed co-expressions since the potential underlying co-regulation between two genes was observed in two different species independently. The evolutionary distance between the species under consideration as well as the availability of experimental data for co-expression detection and orthology inference determine the limits for this approach.

For the comparison of *S. cerevisiae *and *C. elegans *data, conserved co-expressed gene pairs tend to code for members of the same protein complex [6] and such pairs show increased prediction accuracies for *S. cerevisiae *gene interactions [7]. By combining orthologs in several species into "metagenes", co-expressed metagenes were identified and biologically meaningful clusters of metagenes were found [8]. It was also shown that pairs of metagenes coding for interacting proteins had a higher co-expression than those coding for non-interacting proteins [9].

In the work present here, we first introduce a method that determines the biological relevance of gene pair interactions according to biological expert knowledge. Employing our method, we compare four distance measures commonly used in gene co-expression analysis. We evaluate these measures in terms of their ability to detect biologically relevant interactions in the three species *S. cerevisiae*, *D. melanogaster *and *C. elegans*, extending previous work done in *S. cerevisiae *only. We then incorporated the conservation of co-expression where genes were co-expressed not only in a single species but in two or three species simultaneously. Accounting for conserved co-expression enables to identify the strong interactions and to exclude weak or sporadic ones. The framework we present here can be readily applied to make use of the rich model organism data for increased accuracies of protein interaction predictions.

## Results

### Distance measure evaluation

In this first part of our analysis we evaluated four distance measures that are frequently applied for the detection of co-expression between genes: the Pearson correlation [10], the Spearman correlation [11], the Euclidean distance [12], and the mutual information [13]. For each of the three species *S. cerevisiae*, *D. melanogaster *and *C. elegans *(see method's section for details) separately, we calculated the co-expression values, using one of the distance measures at a time, between all pairs of genes and ranked the pairs according to their corresponding distances. To evaluate the accuracy of co-expression predictions, we calculated a function similarity measure that described how well the two genes in a gene pair were associated according to biological expert knowledge. For this, we followed a suggestion by Lord et al. [14] that has previously been used for gene co-expression network analysis [15] and employed the directed acyclic graph (DAG) structure of the Gene Ontology (GO) annotation system [16] (version date: March 24^th^, 2006). In the GO system, a gene can be annotated to more than one functional attribute. For each of the two genes in a pair, we extracted a sub tree of *biological process *annotation attributes (nodes). As the measure of functional similarity between the two genes we then calculated the ratio of the nodes found in both trees (intersection) and compared it to the union of both trees thus defining a similarity measure between 0 for unrelated genes and 1 for genes with identical annotations. The KEGG PATHWAY maps [17] (release 35) were also considered in parallel, to give a second analysis of biological expert knowledge that was independent of the GO annotations. For this, we followed a previously published approach [7] where two genes were said to have similar function if they occurred on the same PATHWAY map.

**Figure 2:**
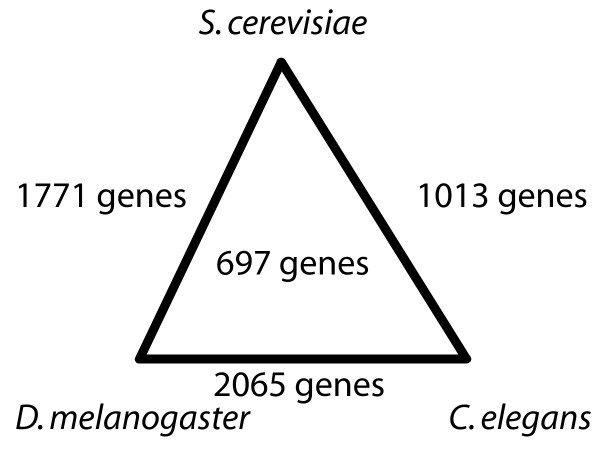
**Sizes of the gene expression datasets used**. We based our analyses on 'synchronized' gene expression datasets for the three species *Saccharomyces cerevisiae *(yeast), *Drosophila melanogaster *(fly), and *Caenorhabditis elegans *(worm) where each gene in one species had a corresponding orthologous gene in one of the other species (number on the edges) or among all three datasets (number inside the triangle).

We used the functional similarities as defined by GO or KEGG to evaluate each gene list that was ranked according to the co-expression between gene pairs (based on 1771 genes for *S. cerevisiae *and 2065 genes for *D. melanogaster *and *C. elegans*, see also Figure 2 and below). For each number of best co-expressed gene pairs (i.e. for 1 to ~8000 gene pairs with the smallest distances), we calculated the average functional similarity of our predictions (which is the same as the accuracy or the positive predictive value). The list of genes that are known not to interact is small and by far incomplete, so it is very difficult to evaluate the sensitivity of our predictions. Instead, we preferred to express the interactions rather in terms of accuracies than as true and false positives in receiver operating characteristics (ROC). Assuming a relation between functional coupling and co-expression, we expected to find the highest accuracy for the best co-expressed genes. The further incorporation of less strongly co-expressed pairs will then lead to a decrease of accuracy. With this accumulative way of evaluation we took the perspective of somebody asking the question similar to: "How well are the 1000 best co-expressed gene associations supported by functional annotations?" or "What is a reasonable number of gene associations I should include to find functionally similar pairs of genes to a certain level of accuracy?"

**Figure 3:**
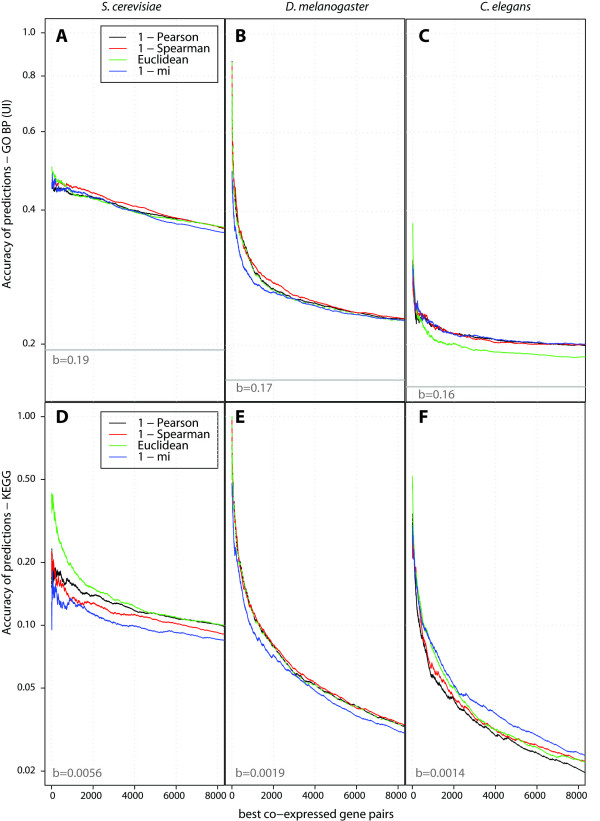
**Evaluation of distance measures**. Four of the most commonly used gene co-expression distance measures were evaluated in terms of their ability to detect biologically relevant gene associations according to *biological process *Gene Ontology annotations (A, B, C) and the KEGG PATHWAY map annotations (D, E, F). We incorporated gene pairs, starting with the highest co-expressed ones, continuing successively with weaker interactions, and predicted their accuracies. The impact of this incorporation on the prediction accuracies for the three species *S. cerevisiae *(yeast) (A, D), *D. melanogaster *(fly) (B, E), and *C. elegans *(worm) (C, F) and for four commonly used distance measures are shown. The background accuracy 'b' (grey horizontal lines) is the average over all gene association and represents the expected accuracy for a randomly chosen gene pairs. For the *S. cerevisiae*, *D. melanogaster*, and *C. elegans *datasets, the analysis is based on 1771, 2065, and 2065 genes, respectively (see also Figure 2). The top 8000 interactions obtained using the Spearman correlation, which was used in the conservation of co-expression study shown in Figure 4, contain 1131, 1308, and 1117 genes for *S. cerevisiae*, *D. melanogaster*, and *C. elegans*, respectively.

At first, we observed for all species and distance measures that the accuracies obtained from GO annotations were generally higher than the accuracies from KEGG maps while the shapes for all graphs were similar: the accuracies decreased continuously as we included more and more of the lower ranked gene pairs (Figure 3). *S. cerevisiae *accuracies decreased less dramatically than the accuracies for the two other species indicating the strong contribution of the even lower ranked gene pairs to the observed averaged accuracy. In terms of overall accuracies, *S. cerevisiae *performed best followed by *D. melanogaster*, while *C. elegans *performed poorest. This order coincided with the overall annotation level (background similarity) for the three species, i.e. the background accuracy was also poorest for *C. elegans *(see also legends in Figure 3). Other factors such as the experimental conditions chosen to generate the expression datasets as well as the overall quality of the datasets might play a role.

A functional GO analysis [18] of the top 8000 genes in *S. cerevisiae *showed several highly significant GO terms related to the ribosome, accounting for 317 genes out of the 1131 genes (28%) in the top 8000 interactions. For the 8000 top *D. melanogaster *interactions, we found overrepresentation of developmental GO terms, which fits well with the experimental conditions of this dataset. For the *C. elegans *dataset, the only two significant GO terms refer to "organelle part".

Only minor performance differences were found between distance measures, and different datasets and species will favor different measures. Euclidean distance and the mutual information were found both as the best and the worst method depending on the situation. The most robust method seems to be Spearman correlation as it was often the best and never the worst method. We noticed that the Euclidean distance has to be handled with care. When we tested the influence of different data normalization schemes (see below) we saw that the Euclidean distance performed poorly when the datasets were not z-normalized (data not shown).

### Conserved co-expression across species

**Figure 4:**
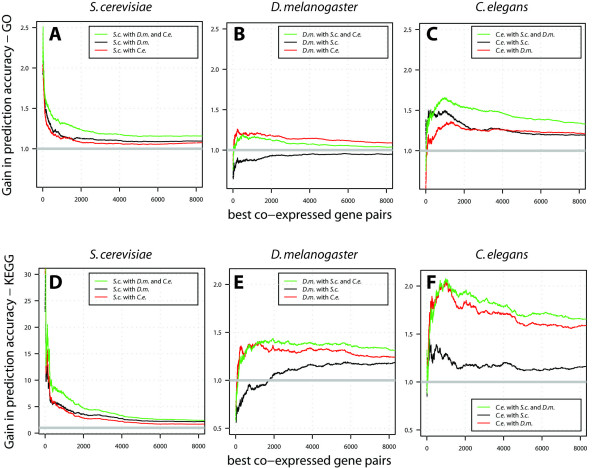
**Function prediction can be improved by combining co-expression of orthologous gene pairs**. The prediction accuracy for co-expressed genes in a species was compared to the accuracies obtained from gene associations that are conserved across two or three species considered in this study. The *gain in accuracy *was obtained as the ratio of the accuracy of the combined co-expression to the accuracy obtained for one species alone. The analysis was based on 697 genes (see also Figure 2) for which orthologous genes were found across all three species.

After we evaluated a set of commonly applied distance measures for co-expression detection for three species separately, we proceeded and asked: "How much gain in accuracy for functional gene interactions do we see when the analysis is restricted to interactions that are conserved between two or even three evolutionary distant species?" For this, we employed the principle of orthology: two genes are orthologous to each other when they arose from a speciation event [19]. A common assumption in this context, even though it is not part of the definition of orthology, is that two orthologs might keep their function partly or even completely (see also method's section for details). The evolutionary distance between two orthologous genes might play a role in so far as orthologs between similar species are generally thought of to retain a higher level of functional conservation than between distant species. We incorporated orthology into our analysis by 'synchronizing' the expression datasets between two species: each gene in one datasets has one corresponding orthologous gene in the other dataset. The number of genes used for the subsequent analysis was thereby reduced compared to the distance measure evaluation performed before (see Figure 2 for the number of genes) because genes with unclear orthology relationships were removed from the analysis. Using the datasets synchronized between two or three species, gene associations between species can be linked to each other. We then combined the distances between two or three species by averaging them (geometric mean gave better results than the arithmetic mean) between the pairs of orthologous genes. The Spearman distance measure was selected for this analysis, as it gave the best overall performance across the datasets (Figure 3). One of the prerequisites here was to normalize the inter-species distances to a common range between zero and one (see method's section for details). For each expression dataset, the impact of incorporating orthologous expression data was evaluated. Similar to the analysis shown in Figure 3, co-expressed gene pairs for each dataset were ranked according to distances calculated from one, two, or three species and each ranked list was evaluated using GO and KEGG functional annotation (Figure 4).

For the *S. cerevisiae *dataset we found that the incorporation of co-expression conservation to the *C. elegans *dataset gave an increase in accuracy and the conservation to the *D. melanogaster *resulted in an even higher increase (Figure 4A and 4D). The joint conservation of *S. cerevisiae *to both other species at the same time increased the accuracy again further, giving a consistent picture for both, GO and KEGG functional annotations.

For *C. elegans*, the simultaneous conservation to both species also outperforms the accuracies obtained when considering only one of the two other species (Figure 4C and 4F). The GO annotation system slightly favors the conservation to *S. cerevisiae *while the situation is inversed for KEGG.

While both *S. cerevisiae *and *C. elegans *benefit positively from considering the conservation to any of the two other species, the incorporation of *S. cerevisiae *could even decrease accuracies below the level of *D. melanogaster *alone. Here we can only speculate about the reasons: The relatively large number of ribosomal gene interactions found for the *S. cerevisiae *dataset (see findings above) might not correspond to highly scoring interactions in the *D. melanogaster *or *C. elegans *dataset, and therefore lead to decreased accuracy. Another explanation is that the information content of the *S. cerevisiae *dataset might be relatively poor so that it benefits from the incorporation of any of the two other datasets. Therefore it only gives little advantage to the *C. elegans *dataset, and even has a negative influence to the *D. melanogaster *dataset. Accounting for the conservation to *C. elegans *increases accuracies so that the combination of *S. cerevisiae *and *C. elegans *still gives an overall gain.

### Influence of dataset and normalization

During our analysis we recognized the strong influence of the normalization scheme on the performance of the distance measures. Specifically for the Euclidean distance we observed an extremely poor performance when expression data was not z-normalized (data not shown). The Pearson correlation and the mutual information were only slightly affected. The Spearman correlation was not affected at all since the rank ordering inherent in this method is invariant to z-normalization.

For *S. cerevisiae*, we also tested the performance of distance measures and their influence on conservation to a second dataset [20] that contained up to 300 experimental conditions. Compared to the Spellman et al. dataset we used in the analysis presented here, the Hughes et al. dataset contained many genes with a higher number of extremely high (and probably biologically not meaningful) expression values (data not shown). Resulting from these outliers, we observed poor performances for the best co-expressed gene pairs. Even after removing these outliers, the Hughes et al. dataset gave less good results in our evaluations so that we decided not to employ it for our analysis.

## Discussion

We have introduced a method to assess the relevance of gene co-expression on the basis of biological expert knowledge. This can be useful for boosting the accuracy of interactions predicted from microarray expression data. This is of particular value for species with limited availability of expression data, given that several organisms already are associated with large amounts of microarray data. In addition to the established binary measure for the co-occurrence of genes on the same KEGG PATHWAY map, we follow a more recent suggestion [14] that utilizes the annotation graphs defined by the Gene Ontology (GO) consortium. In this more fine-grained approach the functional similarity between two genes is a continuous value between zero and one, with higher values representing gene association with higher biological relevance. In our method, we evaluate the gene interactions in a top-down manner, starting with the most co-expressed pair and including each next best co-expressed gene pair one at a time. This way the impact of successively incorporated gene associations becomes apparent and the disadvantages of binning procedures can be avoided. It is particularly useful for revealing whether the strongest co-expressed gene pairs constitute the most promising candidates for experimental assays to detect the functions of uncharacterized genes.

Distance calculations for gene expression profiles are extensively performed, mainly for the clustering of gene expression data [10]. However, the present study is one of the first to systematically evaluate the direct impact of the different distance measures on the detected gene associations for several species. In general, distance measures are chosen without justification or considering the suitability of the species or experimental conditions of the expression data under consideration. Our evaluation of several commonly used distance measures on expression data of three different species draws a fairly consistent picture. Both annotation systems, GO and KEGG, mainly coincided in the results we obtained. Considering the general results over all three species, we neither found a distance measure that underperformed when compared to the other measures nor was one of them clearly outperforming the others. The preprocessing of the gene expression data constituted a crucial parameter for our analysis (data not shown). Here, the Euclidean distance appeared specifically sensitive to outlying values and produced poor results when the data preprocessing was not sufficiently balanced.

We systematically compared the prediction accuracies of gene co-expression obtained from a single species to the accuracies obtained from interactions that are conserved across two or three species [see Additional file 1]. By evaluating the accuracies among gene co-expression that was conserved between all the three species used in this study at the same time, we extended previous investigations in which the impact of conservation has been evaluated on only one species (most often on *S. cerevisiae*) and on broad functional classes (e.g. MIPS complexes) [7].

## Conclusion

By assessing the biological relevance of gene interactions directly, and not via the overrepresentation analyses of annotations for potentially functionally related groups of genes, we were able to systematically analyze various normalization parameters and distance measures. Even though the presented analysis exemplifies the proposed method only with a few species, datasets, and distance measures, it can be applied to evaluate a wide variety of data resources and the impact of various parameter settings.

## Methods

### Gene expression data

We used gene expression data from the three species *Saccharomyces cerevisiae *[21], *Drosophila melanogaster *[22], and *Caenorhabditis elegans *[23]. We normalized the genes for each of the three datasets. For the *D. melanogaster *and the *C. elegans *datasets, we first removed log-ratio expression values that had a distance from the median of more or less than 5 times the inner quartile range. For all three species we then z-normalized the expression values for each gene to a mean of zero and a standard deviation of one.

### Orthology

The orthology database InParanoid [24] (version 4.0) was used to determine the subsets of genes that were shared between each of the three pair wise species comparisons and for the three-way species comparison. For simplicity, we only used the two seed orthologs from each InParanoid ortholog group for the analysis. If a group contained multiple seed orthologs, the first one was taken. Applying this procedure, we obtained different dataset sizes for the pair wise species comparisons and for the three-way comparison (Figure 2).

### Distance measures and normalization

We evaluated a set of commonly applied distances measures in gene expression data analysis. These measures were the Pearson correlation [10], the Spearman correlation [11], the Euclidean distance [12], and the mutual information [13]. Except for the Euclidean distance, which already fulfills the requirements of a distance measure, these measures were transformed into distances by subtracting their absolute value from unity and were then linearly scaled to range between 0 and 1. To reduce the effects arising from undefined data, we only considered distance values where two genes had at least 80% of their expression values simultaneously defined. We estimated the mutual information using the standard histogram procedure [25] and chose appropriate histogram resolutions depending on the number of experimental conditions of each of the datasets (5 bins for the *D. melanogaster *dataset with 70 experimental conditions, 10 bins for the *C. elegans *dataset with up to 548 experimental conditions, and 5 bins for the *S. cerevisiae *dataset with 77 experimental conditions). For the mutual information, we additionally considered the *finite size effect *that describes the systematic overestimation of the mutual information depending on the number of data points from which it is calculated [26]. By correcting for this effect, we avoided biases arising from a different number of undefined values in the gene expression data.

### Significance of accuracies

We estimated the significances of the prediction accuracies with a permutation test. We randomly permuted the ordering of the gene pairs in the list, which was originally obtained using the co-expression distance measures under consideration, and re-calculated the accuracies for the whole range of thresholds. From 1000 random realizations we estimated the mean and the standard deviation of the accuracies. The significances were obtained from the difference of the mean values between original and randomized data in terms of standard deviations of the randomized data and are also referred to as *z-scores*. All accuracies depicted in Figures 3 and 4 were highly significant (p << 1e-04).

## Authors' contributions

COD designed the study, conducted the analysis, and drafted the manuscript. ELLS edited the manuscript. All authors read and approved the final manuscript.

## Supplementary Material

Additional file 1 **Predicted protein-protein interactions**. For each pair of genes used for the distance measure evaluation shown in Figure 4, the co-expression Spearman distance as well as the distances according to gene ontology (GO) and KEGG PATHWAYS are provided. Click here for file
